# Correlation between BRCA mutational status and lymph node metastasis in advanced epithelial ovarian cancer: a retrospective study

**DOI:** 10.7717/peerj.21823

**Published:** 2026-10-20

**Authors:** Mingfei Guan, Xiaojing Wang, Yinping Ma, Qing Wu, Li Zhou, Yan Li, Anna Zhu

**Affiliations:** Department of Gynecological Oncology, Cancer Hospital of Shantou University Medical College, Shantou, Guangdong Province, China

**Keywords:** Epithelial ovarian cancer, BRCA mutation, Lymph node metastasis, Prognosis, Prognostic factors

## Abstract

**Objective:**

To clarify the association between breast cancer (BRCA) mutations and clinically relevant factors, with a particular focus on lymph node metastasis (LNM), in patients with advanced epithelial ovarian cancer (EOC).

**Methods:**

This retrospective single-center study included patients with histologically confirmed advanced EOC and known BRCA status. Comprehensive clinical, surgical, and pathological data were collected, and survival outcomes were obtained through follow-up. Associations between BRCA mutations and clinicopathological variables were analyzed using univariate and multivariate models. Progression-free survival (PFS) and overall survival (OS) were assessed using Kaplan–Meier analysis and Cox proportional hazards regression.

**Results:**

A total of 106 patients were included, of whom 34 (32.1%) harbored BRCA mutations. Univariate analysis demonstrated that BRCA-mutated patients had significantly higher rates of abdominopelvic LNM, distant LNM, cardiophrenic LNM, and distant metastasis, as well as more advanced International Federation of Gynecology and Obstetrics (FIGO) stages and higher baseline CA153 levels (≥ 100 U/ml). Multivariate analysis identified abdominopelvic LNM, distant LNM, and baseline CA153 ≥ 100 U/ml as independent correlates of BRCA mutation status (all *P* < 0.05). Furthermore, for OS, BRCA gene status and achievement of R0 resection were independent prognostic predictors for advanced EOC (both *P* < 0.05); for PFS, elevated HE4 (≥ 500 U/ml) was identified as an independent adverse prognostic factor (HR = 2.292, 95% CI [1.202–4.371], *P* = 0.012).

**Conclusion:**

This study demonstrates that patients with advanced EOC harboring BRCA mutations are more likely to develop both regional and distant LNM. Nevertheless, BRCA mutation status and complete cytoreductive surgery (R0 resection) are associated with favorable prognosis and prolonged survival. Therefore, routine BRCA genetic testing is strongly recommended for all patients with advanced EOC, particularly those presenting with LNM, to facilitate individualized treatment strategies and prognostic assessment.

## Introduction

Epithelial ovarian cancer (EOC) remains the most lethal gynecological malignancy worldwide and continues to pose a substantial threat to women’s health. The global 5-year overall survival rate is estimated to be approximately 40–50% (Bray et al., 2024; Blanc-Durand et al., 2025). The identification of breast cancer (BRCA) genes provided the foundation for precision prevention, early diagnosis, and targeted therapy in ovarian cancer. Previous studies have suggested that BRCA mutational status is associated with distinct clinicopathological characteristics and may influence disease behavior and treatment response (Pan et al., 2025).

In our preliminary clinical observations, patients harboring BRCA mutations exhibited a higher incidence of lymph node metastasis (LNM). To further investigate this association, we conducted a retrospective analysis of patients with advanced EOC who underwent BRCA genetic testing at our institution. The primary objective of this study was to systematically characterize clinicopathological features stratified by BRCA mutation status, with particular emphasis on LNM. We sought to clarify the clinical relevance of BRCA mutations beyond therapeutic decision-making, with the aim of informing genetic counseling, optimizing individualized treatment strategies, and refining prognostic assessment in patients with advanced EOC.

## Materials and Methods

### Patient selection

This retrospective study initially enrolled 286 patients diagnosed with ovarian malignancies who were admitted to the Department of Gynecological Oncology, Cancer Hospital of Shantou University Medical College, between June 2020 and May 2024. Advanced disease was defined as International Federation of Gynecology and Obstetrics (FIGO) stage III–IV. Patients were excluded if they had non-epithelial ovarian tumors, early-stage (FIGO I–II) disease, unknown BRCA status, or incomplete clinical and follow-up data. The final cohort comprised 106 eligible patients with advanced EOC (Fig. 1).

**Figure 1:**
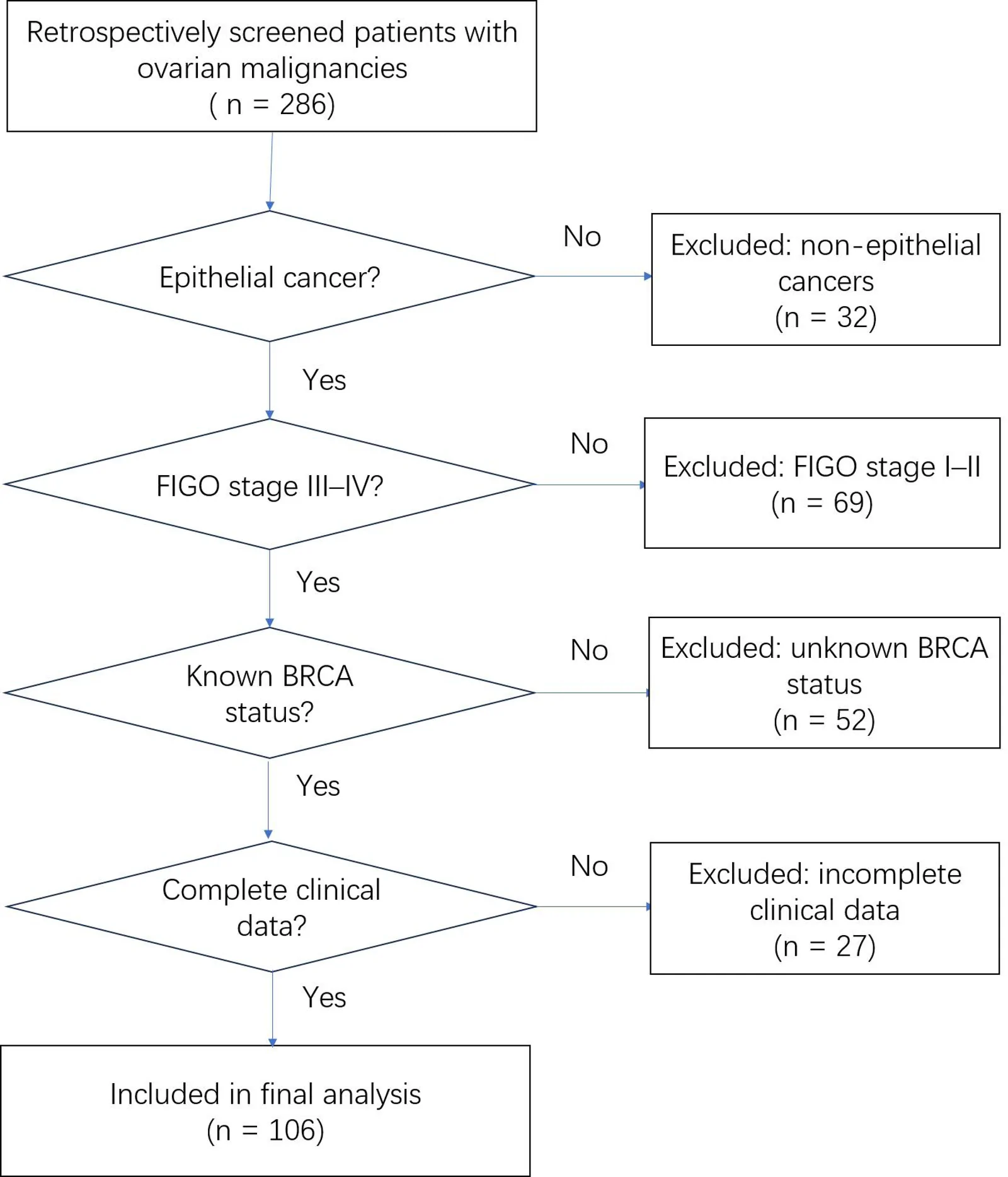
Flowchart of patient selection.

### Clinical data collection

Comprehensive clinical and pathological data were collected for all enrolled patients, including pretreatment tumor markers, disease extent with particular attention to LNM, FIGO staging, use of neoadjuvant chemotherapy (NACT), and surgical resection status.

### Imaging and staging

All patients underwent imaging evaluation of at least the neck, chest, abdomen, and pelvis within two weeks prior to treatment initiation using computed tomography (CT), magnetic resonance imaging (MRI), or positron emission tomography-computed tomography (PET/CT). For patients undergoing primary cytoreductive surgery (PCS), staging was performed according to the 2014 FIGO surgical-pathological staging criteria, based on preoperative imaging findings and surgical pathology reports. Patients who underwent interval cytoreductive surgery (ICS) following NACT were staged using pretreatment imaging findings in accordance with the same criteria.

Radiographic criteria for the diagnosis of LNM were defined as follows (Nougaret et al., 2017): (1) Lymph node size: distant nodes with a short axis > 0.5 cm, or abdominopelvic nodes with a short axis > 1.0 cm; (2) Lymph node morphology: irregular margins, serrated appearance, or indistinct boundaries with adjacent structures; (3) Lymph node density: peripheral enhancement with or without central hypodensity; (4) Confirmation of LNM by PET/CT.

### Treatment regimens

Among the 106 eligible patients, 64 (60.4%) underwent PCS, while 42 (39.6%) received interval cytoreductive surgery following 2–5 cycles of neoadjuvant chemotherapy. All patients subsequently completed 3–6 cycles of adjuvant chemotherapy, resulting in a total of 6–8 cumulative cycles of platinum-based chemotherapy. Cytoreductive surgery was classified as R0 (no gross residual tumor), R1 (residual tumor ≤ one cm), or R2 (residual tumor > one cm). Satisfactory cytoreduction was defined as R0 or R1 resection.

### BRCA genetic testing

All BRCA genetic testing was performed using paraffin-embedded tumor tissues and peripheral blood samples. For genetic analysis, targeted capture sequencing (targeted region probe capture and next-generation sequencing (NGS)) was utilized to analyze circulating tumor DNA (ctDNA) and tumor tissue samples. Testing was conducted at certified laboratories, including the Department of Pathology of the Cancer Hospital of Shantou University Medical College, PRG Benchmark Technology Co., Ltd. (Beijing, China), and Shenzhen BGI Clinical Testing Center.

Genomic DNA was extracted from FFPE tumor tissues using the QIAamp DNA FFPE Tissue Kit, and circulating cell-free DNA (cfDNA) was isolated from 4–5 mL plasma with the QIAamp Circulating Nucleic Acid Kit (Qiagen, Hilden, Germany). Sheared DNA fragments ranging from 200 to 400 bp were purified with Agencourt AMPure XP magnetic beads, hybridized with capture probes, enriched by magnetic separation, and amplified for library construction. Two capture strategies were adopted in this cohort: (1) The commercial OncoScreen Plus panel covering 520 genes across 1.64 megabases of the human genome; (2) An oligonucleotide probe capture assay covering all exons and ± 20 bp flanking sequences of *BRCA1* (RefSeq NM_007294.3) and *BRCA2* (RefSeq NM_000059.3).

Indexed DNA libraries were sequenced on Illumina NovaSeq 6000 and NextSeq 500 platforms with paired-end reads. The mean sequencing depth reached 1,000 × for FFPE tumor tissues and 10,000 × for plasma ctDNA samples. Raw sequencing reads were mapped to the human reference genome hg19 using Burrows-Wheeler Aligner (BWA v0.7.10). Genome Analysis Tool Kit (GATK v3.2) and VarScan v2.4.3 were used for local realignment optimization, duplicate marking, and variant calling. Somatic variants were identified by comparing tumor or ctDNA sequencing data against matched peripheral blood leukocyte controls.

Germline variants were classified according to the American College of Medical Genetics and Genomics (ACMG) guidelines and grouped into five classes as benign (Class 1), likely benign (Class 2), variants of unknown significance (VUS; Class 3), likely pathogenic (Class 4), and pathogenic (Class 5). Somatic variants were interpreted following the Association for Molecular Pathology (AMP), the American Society of Clinical Oncology (ASCO), and the College of American Pathologists (CAP) guidelines and categorized into four grades based on clinical significance: Grade I (variants of high clinical significance); Grade II (variants of potential clinical significance); Grade III (variants of uncertain clinical significance); and Grade IV (benign/probably benign variants). Patients harboring germline pathogenic or likely pathogenic BRCA mutations, or somatic BRCA variants of high or potential clinical significance, were defined as BRCA gene mutation carriers. Cases carrying VUS were excluded from the BRCA-mutant cohort.

### Follow-up

Patient survival status was assessed through telephone follow-up and outpatient visits. The follow-up cutoff date was July 31, 2025. Overall survival (OS) was defined as the interval from initial diagnosis to death from any cause or the date of last follow-up. Progression-free survival (PFS) was defined as the interval from initial diagnosis to the first documentation of disease progression or death from any cause, whichever occurred first. Patients without progression or death were censored at the date of last follow-up.

### Statistical analysis

All statistical analyses were performed using SPSS 27.0 (IBM Corp., Armonk, NY, USA). Continuous variables were presented as mean with standard deviation (SD) for normally distributed data or median with range for non-normally distributed data. Normality of distribution was assessed using the Shapiro–Wilk test. Continuous variables were compared using the independent-samples *t*-test for normally distributed data and the Mann–Whitney U test for non-normally distributed data. Categorical variables were compared using the chi-square test or Fisher’s exact test as appropriate. Fisher’s exact test was employed when the expected frequency in any cell of the contingency table was less than 5. For the analysis of factors associated with BRCA mutation status (binary outcome), multivariate logistic regression models were constructed to identify independent factors associated with BRCA mutations.

For survival analysis, survival rates were estimated using the Kaplan–Meier method. Univariate analysis of survival-related factors was performed using the log-rank test, and variables with *P* < 0.05 were selected as candidates for multivariate analysis. Multivariate survival analysis was performed using Cox proportional hazards regression models to identify independent prognostic factors for PFS and OS. The statistical significance level was set at *α* = 0.05 for all tests (two-tailed).

### Human ethics and consent to participate declarations

The study protocol was reviewed and approved by the ethics committee of the Cancer Hospital of Shantou University Medical College, Shantou, China (2025-II-046), and the study has been granted an exemption from requiring written informed consent by the ethics committee on June 29, 2025.

## Results

### BRCA mutation status and age at diagnosis

Among the 106 eligible patients, 34 (32.1%) were identified as BRCA mutation carriers, including 19 patients with BRCA1 mutations (12 germline, seven somatic) and 15 patients with BRCA2 mutations (nine germline, six somatic). The age of the overall cohort ranged from 31 to 80 years, with a median age of 60 years. BRCA mutation carriers ranged in age from 31 to 72 years (median: 59 years), whereas BRCA wild-type patients ranged from 33 to 80 years (median: 60 years). No statistically significant difference was observed in age distribution between the two groups (*U* = 1,107.5, *P* = 0.43).

Further subgroup analysis showed that patients with BRCA1 mutations had an age range of 31 to 70 years (median: 57 years), while those with BRCA2 mutations ranged from 42 to 72 years (median: 60 years). The difference in age distribution between BRCA1 and BRCA2 mutation carriers was not statistically significant (*U* = 92.5, *P* = 0.083).

### Clinical and pathological characteristics stratified by BRCA status

According to FIGO staging, 74 patients (69.8%) were classified as Stage III, and 32 patients (30.2%) as Stage IV. Among BRCA mutation carriers, 15 patients (44.1%) presented with Stage III disease and 19 patients (55.9%) presented with Stage IV disease. In contrast, among BRCA wild-type patients, 59 patients (81.9%) had Stage III disease and 13 patients (18.1%) had Stage IV disease. BRCA mutation carriers presented with significantly more advanced FIGO stages compared with wild-type patients (*χ*^2^ = 15.680, *P* < 0.001), with a higher proportion of Stage IV disease in the BRCA mutation group (55.9% *vs.* 18.1%).

Histopathological evaluation indicated that the majority of tumors were high-grade serous adenocarcinoma (92 cases, 86.8%), followed by endometrioid adenocarcinoma (10 cases, 9.4%), clear cell carcinoma (two cases, 1.9%), mucinous adenocarcinoma (one case, 0.9%), and carcinosarcoma (one case, 0.9%). Among the 34 BRCA mutation carriers, 31 patients (91.2%) had high-grade serous carcinoma and three patients (8.8%) had endometrioid adenocarcinoma. In contrast, the BRCA wild-type group comprised 61 cases (84.7%) of high-grade serous adenocarcinoma and 11 cases (15.3%) of other pathological types. No statistically significant difference in pathological type distribution was observed between the BRCA mutation and wild-type groups (*P* = 0.541, Fisher’s exact test).

Regarding the use of NACT, 18 BRCA mutation carriers (52.9%) and 46 BRCA wild-type patients (63.9%) underwent primary cytoreductive surgery without NACT, while 16 BRCA mutation carriers (47.1%) and 26 BRCA wild-type patients (36.1%) received NACT. No statistically significant difference in NACT utilization was observed between the two groups (*χ*^2^ = 1.157, *P* = 0.282).

Regarding surgical cytoreduction, 60 patients (56.6%) achieved R0 resection, 23 patients (21.7%) underwent R1 resection, and 23 patients (21.7%) had R2 resection. The R0 resection rate was 62.5% (45/72) in the BRCA wild-type group and 44.1% (15/34) in the BRCA mutation group, with no statistically significant difference between the two groups (*χ*^2^ = 3.277, *P* = 0.194).

### Association between BRCA mutation status and metastatic patterns

Univariate analysis revealed that patients harboring BRCA mutations had a significantly higher incidence of abdominopelvic LNM, distant LNM, cardiophrenic LNM, and distant metastasis compared with BRCA wild-type patients. Additionally, BRCA mutation carriers were more likely to present with advanced FIGO stages and elevated baseline CA153 levels (≥ 100 U/ml). Notably, no significant difference was observed between the BRCA mutation and wild-type cohorts in the proportion of stage IVB patients without distant LNM, suggesting that distant metastasis in BRCA-mutated patients predominantly involved distant lymph nodes rather than visceral organs (Table 1).

**Table 1:** Comparison of clinical and pathological characteristics between BRCA mutation and BRCA wild-type groups.

Clinicopathological factors	BRCA wild-type	BRCA mutation	*χ* ^2^	*P*
Abdominopelvic LNM^a^			18.611	0.000^*^
No	45	6		
Yes	27	28		
Distant LNM			18.120	0.000^*^
No	62	16		
Yes	10	18		
Cardiophrenic LNM				0.002^*^
No	69	25		
Yes	3	9		
Pelvic peritoneal metastasis				0.464
No	5	4		
Yes	67	30		
Abdominal peritoneal metastasis			0.231	0.631
No	18	10		
Yes	54	24		
Distant metastasis^b^			13.583	0.000^*^
No	59	16		
Yes	13	18		
Distant metastasis excluding LNM^c^				0.678
No	68	31		
Yes	4	3		
CA125			2.189	0.139
<500 U/ml	25	7		
≥500 U/ml	47	27		
HE4			0.100	0.751
<500 U/ml ≥500 U/ml	32	14		
	40	20		
CA153			8.413	0.004^*^
<100 U/ml ≥100 U/ml	47	12		
	25	22		

**Notes.**

a LNM, Lymph node metastasis.

b Distant metastasis refers to stage IVB patients.

c Distant metastasis excluding LNM refers to stage IVB patients without distant lymph node metastasis; Categorical variables were compared using the chi-square test or Fisher’s exact test as appropriate.

* Indicates *P* value < 0.05.

Variables that were significant in univariate analysis were entered into a multivariate logistic regression model, showing that abdominopelvic LNM, distant LNM, and baseline CA153 ≥ 100 U/ml emerged as independent predictors of BRCA mutation status, as shown in Table 2.

**Table 2:** Multivariate analysis of factors associated with BRCA gene mutation.

	*B*	*SE*	*Wald*	*P*	*OR (95% CI)*
Abdominopelvic LNM	1.502	0.562	7.140	0.008	4.492 (1.492–13.522)
Distant LNM	1.290	0.546	5.584	0.018	3.634 (1.246–10.599)
CA153 ≥100 U/ml	1.070	0.492	4.721	0.030	2.914 (1.110–7.648)

**Notes.**

B regression coefficient SE standard error OR odds ratio CI confidence interval LNM lymph node metastasis

This logistic regression model assesses factors associated with BRCA mutation status (binary outcome). Variables with *P* < 0.05 in univariate analysis were entered into the multivariate logistic regression model.

### Survival outcomes and prognostic factors

The median follow-up duration for PFS was 23 months (range: 5–61 months), with a progression-free rate of 51.9% at the last follow-up. Univariate survival analysis using the log-rank test identified pathological type, abdominal peritoneal metastasis, pelvic peritoneal metastasis, R0 resection, and baseline HE4 levels as prognostic factors for PFS (Table 3). Multivariate Cox regression analysis demonstrated that elevated HE4 (≥ 500 U/ml) was an independent adverse prognostic factor for PFS (HR = 2.292, 95% CI [1.202–4.371], *P* = 0.012), while the remaining variables were not independently associated with PFS.

**Table 3:** Univariate analysis of PFS in advanced epithelial ovarian cancers.

	Group	Cases	Events	PFS (%)	*χ* ^2^	*P*
Pathological type	HGSOC	92	48	47.8	4.198	0.040
	Others^a^	14	3	78.6		
Abdominal peritoneal metastasis	No	28	9	67.9	4.479	0.034
	Yes	78	42	46.2		
Pelvic peritoneal metastasis	No	9	1	88.9	4.090	0.043
	Yes	97	50	48.5		
R0	No	46	28	39.1	6.537	0.011
	Yes	60	23	61.7		
HE4	<500 U/ml	46	16	65.2	8.576	0.003
	≥500 U/ml	60	35	41.7		
CA125	<500 U/ml	32	11	65.6	2.393	0.122
	≥500 U/ml	74	40	45.9		
BRCA mutation	No	72	36	50.0	1.732	0.188
	Yes	34	15	55.9		

**Notes.**

PFS Progression-free survival Events number of patients who experienced disease progression or death during the follow-up period PFS (%) percentage of patients without disease progression at the last follow-up date HGSOC High-grade serous ovarian cancer

a Other types include mucinous adenocarcinoma, clear cell carcinoma, endometrioid carcinoma, and carcinosarcoma; *P* values were calculated using the log-rank test.

The median follow-up duration for OS was 33 months (range: 11–61 months), with an overall 3-year survival rate of 77.4%. Univariate survival analysis identified abdominopelvic LNM, surgical resection status (R0), BRCA mutation status, and baseline CA153 levels as prognostic factors for overall survival in patients with advanced EOC (Table 4). Multivariate analysis further demonstrated that R0 resection (HR = 0.270, 95% CI [0.104–0.699], *P* = 0.007) and BRCA mutation (HR = 0.177, 95% CI [0.035–0.894], *P* = 0.036) were independent protective factors for advanced EOC.

**Table 4:** Univariate analysis of OS in advanced epithelial ovarian cancers.

	Group	Cases	Events	Survival (%)	*χ* ^2^	*P*
Abdominopelvic LNM	No	51	14	72.5	4.180	0.041
	Yes	55	7	87.3		
R0	No	46	14	69.6	3.964	0.046
	Yes	60	7	88.3		
BRCA mutation	No	72	19	73.6	9.773	0.002
	Yes	34	2	94.1		
CA153	<100 U/ml	59	15	74.6	4.223	0.040
	≥100 U/ml	47	6	87.2		
CA125	<500 U/ml	32	8	75.0	1.787	0.181
	≥500 U/ml	74	13	82.4		

**Notes.**

OS Overall survival Events number of deaths during the follow-up period Survival (%) survival rate at the last follow-up date LNM lymph node metastasis

*P* values were calculated using the log-rank test.

Patients with BRCA mutations exhibited significantly improved survival outcomes compared with BRCA wild-type patients, with 3-year OS rates of 95.0% and 69.0%, respectively. Kaplan–Meier survival curves are presented in Fig. 2. Similarly, patients with R0 resection had a markedly higher 3-year OS rate than those with residual disease (87.1% *vs.* 68.7%). Kaplan–Meier survival curves are presented in Fig. 3.

**Figure 2:**
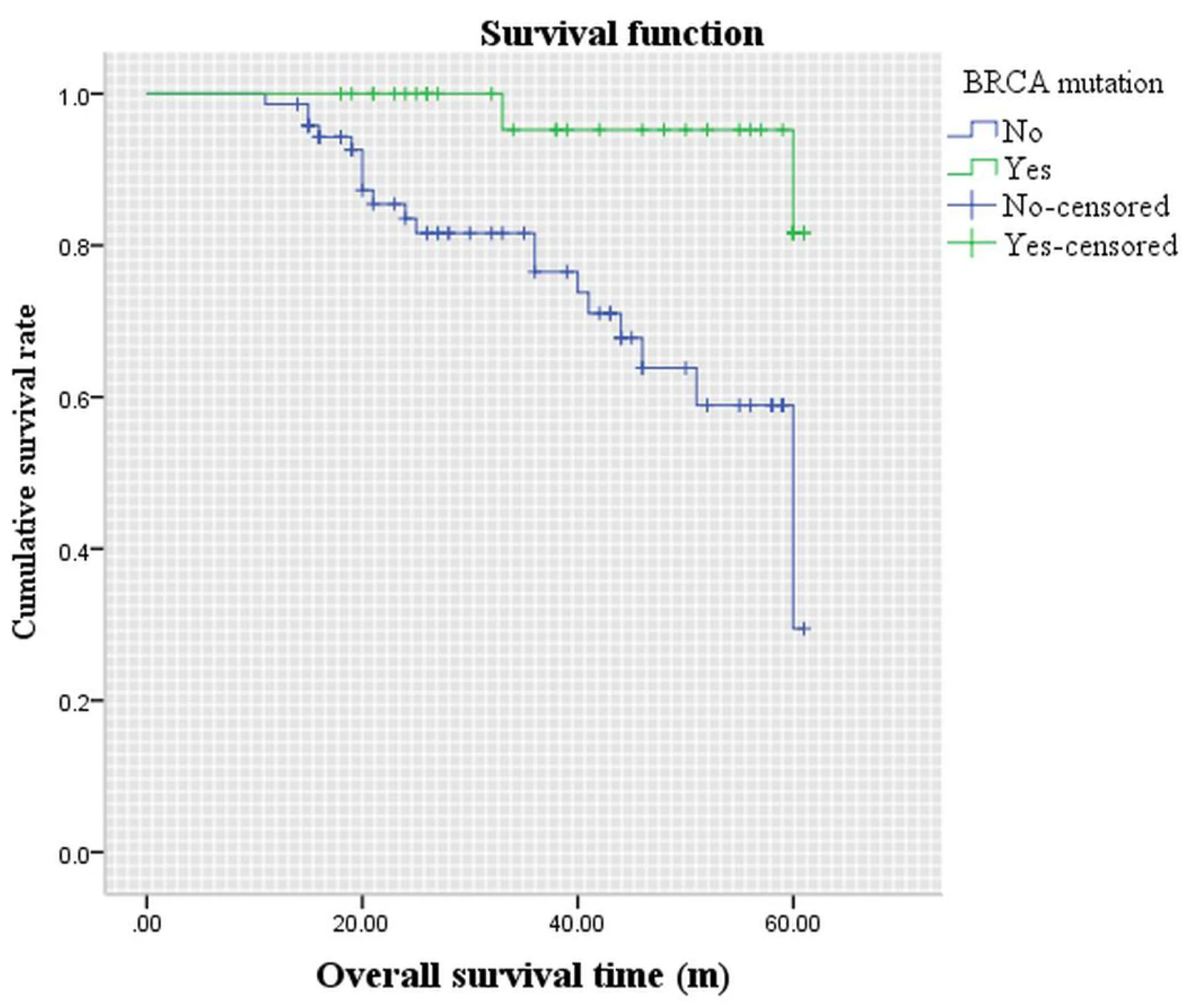
Kaplan–Meier survival curves of patients with BRCA mutation.

**Figure 3:**
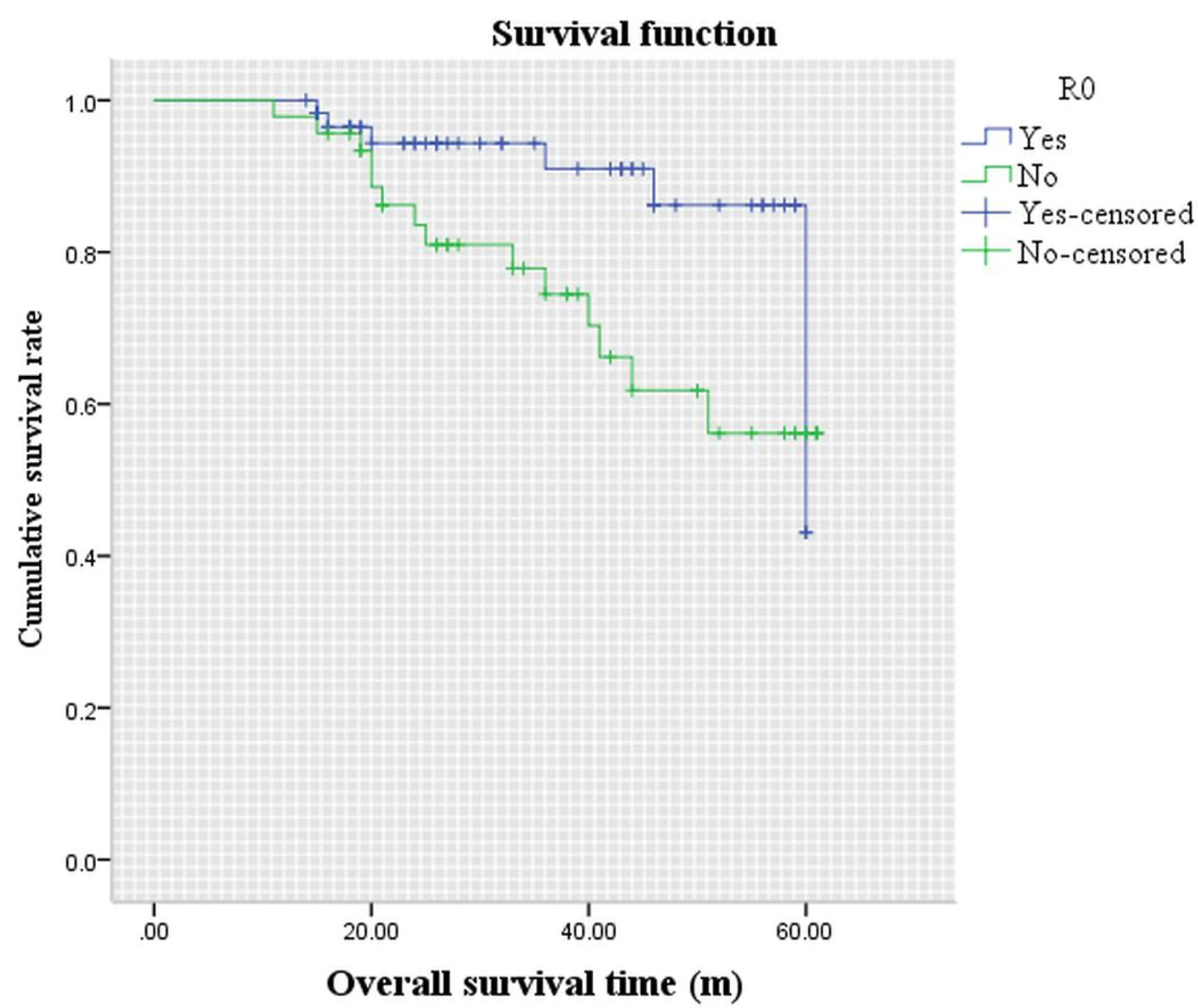
Kaplan–Meier survival curves of patients with R0 resection.

## Discussion

### Incidence of BRCA gene mutations

In this retrospective cohort, the prevalence of BRCA mutations among patients with advanced EOC was 32.1%, which is comparable to previously reported rates in Chinese populations (Yuan et al., 2023). Notably, BRCA mutation prevalence varies substantially across ethnicities and geographic regions. Lower mutation frequencies have been reported in the Brazilian population (12.5%) (Richau et al., 2024), compared with cohorts from the United States (17.5%) (Norquist et al., 2016) and the African American population (21.5%) (Somasegar et al., 2021). A relatively higher prevalence of BRCA mutations has been documented in Chinese populations (Yuan et al., 2023), with even higher rates reported among the Italian population (34.3%) (Tortorella et al., 2024).

### Relationship between BRCA mutations and age at diagnosis

In the present study, no significant difference in age at diagnosis was observed between BRCA mutation carriers and BRCA wild-type individuals. Among BRCA mutation carriers, patients with BRCA1 mutations tended to be slightly younger than those with BRCA2 mutations (median age, 57 *vs.* 60 years), although this difference was not statistically significant (U = 92.5, *P* = 0.083). This trend is consistent with a prior large-scale study (Cummings et al., 2021) demonstrating that ovarian cancer associated with BRCA1 mutations tends to occur at an earlier age than BRCA2-associated disease. Consequently, the National Comprehensive Cancer Network (NCCN) guidelines recommend earlier prophylactic bilateral salpingo-oophorectomy for BRCA1 mutation carriers (35–40 years) compared to BRCA2 mutation carriers (40–45 years) (Daly et al., 2023).

### Association between BRCA gene mutations and lymph node metastasis

This study was motivated by clinical observations suggesting a potential association between BRCA mutation status and LNM. Our findings confirmed that both regional abdominopelvic LNM and distant LNM—particularly cardiophrenic and supraclavicular lymph nodes—were more frequent among BRCA mutation carriers. With the increasing adoption of systemic R0 resection in ovarian cancer in recent years, cardiophrenic LNM has gained greater clinical relevance. Our data indicate that BRCA mutation carriers also exhibit a higher rate of metastasis to these sites.

These findings are consistent with previous studies. For instance, Deng et al. (2019) demonstrated that BRCA1 and BRCA2 mutation carriers were more likely to develop lymph node metastases, and that BRCA mutation status outperformed serum biomarkers in predicting nodal involvement. Similarly, Li et al. (2021) reported a higher incidence of supradiaphragmatic LNM in BRCA-mutated ovarian cancer patients.

The association between BRCA mutations and LNM has also been documented in breast cancer. Chen et al. (2020) observed that in invasive breast cancer, BRCA2 mutation carriers exhibited a higher rate of LNM. Factors associated with BRCA1 mutations include breast cancer diagnosis before the age of 40, tumor size > 2 cm, and LNM (Huang et al., 2022).

Given that tumors from BRCA-mutated women harbor higher tumor-infiltrating lymphocyte (TIL) counts, the immune microenvironment may be significantly influenced by BRCA mutation status, ultimately contributing to distinct disease characteristics (Petrillo et al., 2017). Low expression of BRCA in ovarian cancer tissue may contribute to the initiation and progression of the disease; it correlates closely with FIGO staging, histological differentiation, and LNM, reflecting disease progression in ovarian cancer patients (Pan et al., 2025). Mutations in the BRCA gene within ovarian cancer tissue result in the production of non-functional proteins, leading to impaired DNA damage repair. This induces cellular degeneration and malignant transformation, facilitating invasive behaviors such as LNM.

### Prognostic implications in BRCA mutation status

In the present study, BRCA mutation status was not identified as a significant prognostic factor for PFS in univariate analysis (*P* = 0.188, Table 3). Although BRCA-mutated patients showed a numerically higher progression-free rate at the last follow-up (55.9% *vs.* 50.0%), this difference did not reach statistical significance. In contrast, multivariate Cox regression identified BRCA mutation status as an independent protective factor for OS (HR = 0.177, 95% CI [0.035–0.894], *P* = 0.036), with BRCA-mutated patients exhibiting a 3-year OS rate of 95.0% compared with 69.0% in wild-type patients. This pattern, in which PFS does not reach significance while OS demonstrates a clear advantage, aligns with the findings of Bu et al. (2019), who reported no significant difference in PFS between BRCA mutation carriers and wild-type patients (14.31 months *vs.* 13.62 months) but observed higher 3-year and 5-year survival rates. However, it is noteworthy that a substantial body of literature has reported that BRCA mutations confer simultaneous benefits in both PFS and OS. A Japanese study revealed that in advanced ovarian cancer, the germline BRCA1/2 pathogenic variant cohort had superior PFS and OS compared with the wild-type group (Abe et al., 2022). An Australian analysis of 1,001 non-mucinous epithelial ovarian cancers demonstrated that germline BRCA mutations are independent predictors of both PFS and OS, with the mutation group showing higher response rates to platinum-based chemotherapy (Alsop et al., 2012). A meta-analysis similarly showed that ovarian cancer patients harboring BRCA1/2 mutations derived greater benefit from platinum-based therapy, exhibiting superior PFS and OS (Shao et al., 2021). Among patients with high-grade serous ovarian cancer, those harboring pathogenic BRCA1 and BRCA2 tumor mutations exhibited better prognosis and longer survival than those without pathogenic BRCA1/2 mutations (Sutrisno et al., 2024). The GOG-218 trial further revealed that BRCA/homologous recombination repair (HRR)-mutated tumors had a lower risk of progression and mortality (Norquist et al., 2017).

The survival advantage observed in BRCA-mutated patients is primarily attributable to the mechanism of synthetic lethality. BRCA1 and BRCA2 proteins play essential roles in the HRR pathway, which maintains genomic integrity by accurately repairing DNA double-strand breaks. Loss-of-function mutations in these genes result in homologous recombination deficiency (HRD), rendering tumor cells exquisitely sensitive to DNA-damaging agents such as platinum-based chemotherapy and poly (ADP-ribose) polymerase (PARP) inhibitors (Evans & Matulonis, 2017). Lord & Ashworth (2017) elegantly described this synthetic lethality paradigm in the clinic, providing the rationale for PARP inhibitor use in BRCA-mutated tumors. Recent evidence further supports the notion that BRCA loss of function, including BRCA1 promoter methylation, is specifically associated with platinum hypersensitivity and favorable clinical outcomes in high-grade ovarian cancer, whereas BRCA-unrelated HRD positivity was not associated with improved platinum sensitivity (Fiegl et al., 2024).

The lack of PFS significance in our study may be attributed to several factors: first, the relatively short median PFS follow-up duration of 23 months may have been insufficient to detect significant differences; second, the proportion of patients who had experienced disease progression remained below 50%, thereby limiting the statistical power to detect significant differences; third, due to financial constraints, a considerable proportion of patients did not receive PARP inhibitors in combination with their initial treatment, but only received PARP inhibitors after disease recurrence, which may be the main reason for the lack of PFS difference and the observed OS benefit in this study. An intriguing paradox emerges from our findings: BRCA-mutated patients exhibited a significantly higher incidence of both regional and distant LNM, as well as more advanced FIGO stages, yet demonstrated markedly superior OS outcomes. This apparent contradiction can be explained by the interplay between “intrinsic biological aggressiveness” and “extrinsic therapeutic sensitivity.” Although BRCA-deficient tumors possess a more aggressive metastatic phenotype, possibly driven by impaired DNA repair facilitating invasive behaviors, their heightened sensitivity to platinum-based chemotherapy and PARP inhibitors counterbalances this inherent aggressiveness during initial treatment. During the primary treatment phase, therapeutic efficacy masks the tumor’s intrinsic high-aggressiveness potential, manifesting as a net survival benefit for BRCA-mutated patients. This paradigm is well-documented in the ovarian cancer literature, where BRCA-mutated tumors consistently demonstrate higher response rates to platinum-based chemotherapy and PARP inhibitors despite their more advanced disease presentation (Evans & Matulonis, 2017).

However, this survival advantage may not persist indefinitely. As the disease progresses and recurrent ovarian cancer undergoes multiple lines of chemotherapy, the “extrinsic therapeutic sensitivity” gradually diminishes while the “intrinsic biological aggressiveness” becomes increasingly dominant, potentially reversing the survival dynamic. Candido-dos Reis et al. (2015) reported that BRCA-mutated patients have a significant short-term survival advantage, but this benefit diminishes over time. Notably, BRCA1-positive patients may ultimately experience a reversal of their survival advantage after five years. A meta-analysis by Nahshon et al. (2022) further confirmed that while the expected higher 5-year survival rate was observed in BRCA-mutated patients, this advantage decreased at 10 years. Among patients who had already survived 5 years, no significant advantage in the probability of surviving an additional 5 years was observed for the BRCA-mutated group. These findings underscore the importance of long-term surveillance and the potential role of extended maintenance therapy in preserving the survival benefit for BRCA-mutated patients.

### Additional prognostic factors in advanced EOC

Regarding PFS, multivariate Cox regression identified elevated HE4 (≥ 500 U/ml) as an independent adverse prognostic factor for advanced EOC (HR = 2.292, 95% CI [1.202–4.371], *P* = 0.012). HE4 is a serum biomarker that has gained increasing attention in ovarian cancer, with multiple studies confirming its association with disease progression and recurrence. In a Korean study of 423 patients, Lee et al. (2024) demonstrated that HE4 normalization after debulking surgery and chemotherapy was significantly associated with improved PFS and OS, regardless of BRCA mutational status. Notably, among patients with BRCA mutations, normalizing HE4 alone yielded higher specificity for predicting 1-year recurrence-free survival than CA125 normalization, suggesting that HE4 may be a particularly valuable prognostic marker in this subgroup. Zhao et al. (2026) further evaluated the prognostic value of HE4 in 97 patients with advanced EOC, and found that elevated preoperative HE4 was independently associated with shorter PFS. HE4 demonstrated relatively higher specificity for predicting recurrence compared with CA125. These findings collectively reinforce the clinical value of HE4 as a prognostic tool for PFS assessment and highlight its potential role in post-treatment surveillance and risk stratification for disease recurrence.

Beyond BRCA gene status, another independent OS prognostic factor for advanced EOC is whether patients achieve R0 resection during cytoreductive surgery. Among all patients, the proportions of R0 resection, R1 resection, and R2 resection were 56.6%, 21.7%, and 21.7%, respectively. More complete cytoreduction was associated with improved patient prognosis. Data from the TRUST study (Mahner et al., 2025) indicated that patients who achieved satisfactory cytoreduction after PCS had the most favorable prognosis, with a median PFS of 27.9 months and a median OS of 67.0 months. Thus, satisfactory cytoreductive surgery, particularly R0 resection (defined as the absence of macroscopically visible residual tumors), is a pivotal prognostic factor for ovarian cancer and represents the ultimate goal pursued by gynecologic oncologists.

## Study limitations

This study has several limitations. First, its retrospective, relatively small sample size and single-center design may limit the generalizability of the findings. Second, detailed information regarding specific BRCA mutation sites was unavailable for some patients, precluding more refined genotype-phenotype analyses. Third, our hospital only launched routine BRCA testing in 2020, resulting in limited follow-up (median 33 months, range: 11–61 months). Given the relatively short follow-up period, median PFS/OS and 5-year survival rates could not be reliably estimated. Therefore, 3-year OS was used as the primary survival endpoint. To address these limitations, future large-scale, prospective, multicenter studies with larger cohorts, comprehensive genomic characterization, and extended follow-up are warranted to validate and extend these observations, thereby strengthening the evidence base for clinical decision-making.

## Conclusion

In conclusion, patients with advanced EOC harboring BRCA mutations are more likely to develop both regional and distant LNM. Nevertheless, BRCA mutation status and achievement of R0 cytoreductive surgery are independently associated with improved survival outcomes. These findings support routine BRCA genetic testing in patients with advanced EOC, particularly those presenting with LNM, to facilitate individualized treatment strategies and optimize prognostic assessment.

##  Supplemental Information

10.7717/peerj.21823/supp-1 Supplemental Information 1 Raw data

10.7717/peerj.21823/supp-2 Supplemental Information 2 Codebook

10.7717/peerj.21823/supp-3 Supplemental Information 3 Univariate analyses

10.7717/peerj.21823/supp-4 Supplemental Information 4 Multivariate logistic regression models

10.7717/peerj.21823/supp-5 Supplemental Information 5 Survival analysis

10.7717/peerj.21823/supp-6 Supplemental Information 6 PFS analysis

10.7717/peerj.21823/supp-7 Supplemental Information 7 Translation codebook for raw data .sav file

## Additional Information and Declarations

### Data Availability

The following information was supplied regarding data availability: The raw measurements are available in the Supplemental Files.
