# Supplementary material for: Correlation between BRCA mutational status and lymph node metastasis in advanced epithelial ovarian cancer: a retrospective study
Source: PeerJ. 2026 Oct 20;14:e21823. doi: 10.7717/peerj.21823 (PMC13693603; doi:10.7717/peerj.21823)
Supplement: Supplementary file 1 [file peerj-14-21823-s002.docx]

Pathological type: 1=High-grade serous ovarian cancer；2=Others

Abdominopelvic LNM: 1=No；2=Yes

Distant LNM: 1=No；2=Yes

Cardiophrenic LNM: 1=No；2=Yes

Abdominal peritoneal metastasis: 1=No；2=Yes

Pelvic peritoneal metastasis: 1=No；2=Yes

Distant metastasis: 1=No；2=Yes

Distant metastasis excluding LNM: 1=No；2=Yes

R0: 1=No；2=Yes

Survival status: 1=Alive；2=Dead

BRCA: 1=Negative；2=Positive

CA125: 1=<500U/ml；2=≥500 U/ml

HE4: 1=<500pmol/L；2=≥500 pmol/L

CA153: 1=<100 U/ml；2=≥100 U/ml

NACT: 1=No；2=Yes

FIGO stage: 1=III；2=IV

Progression status: 1=no progression；2=Progression

(LNM: lymph node metastasis)
