# Supplementary material for: Correlation between BRCA mutational status and lymph node metastasis in advanced epithelial ovarian cancer: a retrospective study
Source: PeerJ. 2026 Oct 20;14:e21823. doi: 10.7717/peerj.21823 (PMC13693603; doi:10.7717/peerj.21823)
Supplement: Supplementary file 2 [file peerj-14-21823-s007.docx]

The non-English characters come from the original Chinese Windows file path stored within SPSS files. No Chinese text exists in the research data values.

Original Chinese path:

E:\自己书写的SCI文章\卵巢癌基因检测\文章\文章上传版本\PeerJ\上传原始数据\英文版\raw data.sav

Translated English path:

E:\My own SCI manuscripts\Ovarian cancer genetic testing\Manuscripts\Manuscript submission version\PeerJ\Upload raw data\English version\raw data.sav
